# Enhancer functions in three dimensions: beyond the flat world perspective

**DOI:** 10.12688/f1000research.13842.1

**Published:** 2018-05-30

**Authors:** Anita Göndör, Rolf Ohlsson

**Affiliations:** 1 Department of Oncology and Pathology, Karolinska Institutet, Karolinska University Hospital, Stockholm, Sweden

**Keywords:** Chromatin, enhancers, 3D, nuclear architecture, gene gating

## Abstract

Transcriptional enhancers constitute a subclass of regulatory elements that facilitate transcription. Such regions are generally organized by short stretches of DNA enriched in transcription factor-binding sites but also can include very large regions containing clusters of enhancers, termed super-enhancers. These regions increase the probability or the rate (or both) of transcription generally in *cis* and sometimes over very long distances by altering chromatin states and the activity of Pol II machinery at promoters. Although enhancers were discovered almost four decades ago, their inner workings remain enigmatic. One important opening into the underlying principle has been provided by observations that enhancers make physical contacts with their target promoters to facilitate the loading of the RNA polymerase complex. However, very little is known about how such chromatin loops are regulated and how they govern transcription in the three-dimensional context of the nuclear architecture. Here, we present current themes of how enhancers may boost gene expression in three dimensions and we identify currently unresolved key questions.

## Introduction

Enhancers of transcription were discovered already in 1981, a time when not much was known about basic principles of transcriptional regulation. For an in-depth review on the discovery of enhancers, see reference ^1^. Since then, it has been demonstrated that enhancers play pivotal roles in the regulation of normal cell physiology and maturation programs. Today, it is estimated, based on high-throughput analyses of chromatin immunopurified DNA (ChIP-seq), that up to a million different enhancers are distributed in the mammalian genome and positioned either proximal or distal to their target promoters ^2– 4^. This information provides two important clues: First, given the estimate of the number of genes, the implication is that most genes are surrounded by more than one enhancer, raising the question of whether such enhancers collaborate or act alone ^3^. Second, the clustering of genes poses a logistic problem for enhancers to boost transcription from specific sets of neighboring genes during development, for example. We now know that the activity of both enhancers and promoters is subject to regulation by chromatin marks, which is discussed in more detail below. The laying down of such chromatin marks by so-called pioneer factors ^5^ needs to be coordinated to enable the correct set of genes and their enhancers to make contact during differentiation. The observation that some enhancers can activate genes more than a million base pairs apart ^2– 4^ also prompted the realization that enhancers operate in three dimensions (3D) to enable their juxtaposition to their distal target genes ^3, 4, 6^. In this way, long-range acting enhancers can paradoxically be in even closer physical proximity to target promoters than more proximal enhancer regions. Rather than providing an extensive overview of different types of enhancer functions, we focus here on recent key observations and questions associated with how the enigmatic encounters between enhancers and promoters promote expression of genes in 3D. This aim is fueled by the realization that we will likely not fully understand enhancer mechanisms without incorporating their mode of action in the 3D context of the nuclear architecture. Therefore, we have organized this overview with a flow of themes converging on novel considerations of how the nuclear architecture and genome organization conspire to increase the specificity and efficiency of transcriptional activation. For more general overviews and images of enhancer functions, please consult references ^3, 7, 8^.

## Enhancers – the chromatin mark connection

Though not yet fully understood, transcriptional enhancer elements have been conceptually linked either with promoting the probability of transcriptional initiation or by increasing the rate of transcriptional elongation ^9^. Independently of whether these different perspectives of enhancer function relate to different types of enhancers or reflect enhancer-promoter proximities or both, enhancers have to overcome the by-default poor affinity between RNA polymerase complexes and promoters ^3^. Although promoters have to acquire chromatin marks such as H3K4me3 and H3K27ac to become active, this per se is not sufficient to trigger transcriptional initiation ^4, 10^. Instead, a system has evolved in eukaryotes with enhancer regions mediating the loading of RNA polymerases to their distal target promoters via chromatin looping. This was first documented in an experimental system using a protein bridge to connect the SV40 enhancer to a target β-globin promoter in *trans* ^11^. For endogenous enhancers, this is a very complicated process that depends on whether the active enhancers have acquired chromatin marks, such as H3K4me1 and H3K27ac, to recruit/render them accessible to clusters of key transcription factors ^4^ and hence long-range interactions ^12^. These in turn interact with the mediator complex, which is made up of multiple factors and, as the name implies, mediates the connection between the primary chromatin fiber and the RNA polymerase complex ^13^.

Physical interactions between active enhancers and promoters generally involve a collaboration between the mediator and cohesin complexes ^14^ to form chromatin loops ^15^. This process is perceived to enable the presentation of the RNA polymerase complex to promoters, which have been marked to become active ^15, 16^. However, the formation of such chromatin loops is not sufficient to activate genes by itself ^8^. The reason is that the large subunit of the RNA polymerase complex recruited to the promoter needs a post-translational modification at serine 2, carried out by protein kinases, such as cyclin-dependent kinases (CDKs) 7–9 ^13^, to overcome inhibition of transcriptional elongation. Strikingly, a large fraction of transcriptionally inactive genes have polymerases loaded onto their promoters during development ^17^. The advantage of this arrangement presumably reflects that any rate-limiting, enhancer-mediated loading of RNA polymerases to target genes is bypassed to facilitate rapid activation kinetics in response to emerging environmental cues during a biological process to coordinate transcription ^18^. However, recent observations suggest that the distinction between promoters and enhancers can be blurred, as some promoters can act as enhancers for other promoters and vice versa ^19^. Given the close biochemical structure of the H3K4me3-marked promoters and H3K4me1 modifications characterizing enhancers, it is conceivable that relatively simple enzymatic reactions ^4^ can regulate switches between promoter and enhancer functions.

## Not all active enhancers have known chromatin marks

As a caveat to this discussion, a screen of the current literature has revealed the existence of enhancer regions apparently devoid of the typical enhancer-specific chromatin marks ^10^. There are at least two issues linked with this deduction: First, we may not have a complete catalogue of chromatin marks, or indeed combinations thereof, involved in enhancer function. Second, current technologies, such as ChIP-seq and Hi-C ^20^, do not have sufficient sensitivity or resolution (or both) to simultaneously measure chromatin states and folding in single cells in relation to a transcriptional process. Both approaches generally use large cell populations that are likely heterogeneous because of differences in cell cycle stages or cell state composition (or both) to obscure the directness of such relationships. Thus, we cannot rule out the existence of dynamic and transient chromatin marks underlying stochastic enhancer-gene interactions that are not picked up by current technologies. Therefore, a more unifying feature underlying the formation of enhancer-promoter complexes posits that known or unknown factors (or both) collaborate in 3D to increase the potential for their mobility and subsequent affinity to each other. This scenario is reinforced by the recent demonstrations that such enhancer-promoter interactions can be influenced by mutually exclusive mechanisms involving either cohesin complexes ^14^ or paired YY1-YY1 interactions ^21^. Time will tell us whether there are many other variations on this theme.

## Enhancer functions in three dimensions and chromatin mobility – the DNA repair connection

There are numerous challenges associated with the loading of an RNA polymerase complex to promoters and their release from promoter proximal pausing, especially when considering that enhancer-promoter interactions can occur over millions of kilobase pairs ^8^. For one, the chromatin fibers likely require release from any topological constraints to facilitate physical encounters between enhancers and promoters. In one early study, this issue was highlighted by the large-scale unfolding of a chromosomal region, targeted by strong viral transcriptional activators, to sample one third of the mammalian nuclear space within an hour ^22^. More recently, it was demonstrated that synchronization of circadian gene transcription by external time cues involves dynamic chromatin movements to and from the repressive environment of the nuclear periphery ^23^. Such events are likely regulated directly or indirectly by factors that influence chromatin compaction and mobility. Interestingly, oscillations in 3D genome organization required circadian complex formation between CTCF, which is associated with many enhancers, and PARP1, which is associated primarily with active promoters ^24^ and is involved in DNA repair ^24^. Knocking down either of these factors thus inhibited the ability of circadian genes to approach the nuclear periphery and counteracted the synchronization of transcriptional oscillations ^23^.

Dynamic changes in chromatin structure, mobility, and the unwinding of the double helix during transcription likely all increase torsional stress that has to be released via factors that regulate DNA topology and repair ^25– 29^. Indeed, binding of the catalytically engaged topoisomerase (TOP) 1 to enhancers and gene bodies and TOP2 to promoters has been described at several genes in response to environmental signals ^25^. Current models of TOP action thus posit that signal-regulated gene expression requires the transient generation of single- and double-strand DNA breaks at regulatory elements, followed by the recruitment of DNA repair machinery to safeguard genomic integrity ^25^. Interestingly, MRE11, which is a repair factor capable of removing covalent TOP1-DNA intermediates, is rapidly recruited to androgen-responsive enhancers ^30^. Moreover, while recruitment of MRE11 facilitates the subsequent binding of ATR, a sensor of single-strand breaks, both MRE11 and ATR are required for the expression of the vast majority of androgen-induced enhancer-derived non-coding RNAs (eRNAs) and for the expression of numerous androgen-induced genes ^30^. These findings point to a role of “programmed” DNA breaks and DNA repair factors beyond the safe regulation of torsional stress during transcription ^25^. A clue to their potential function might be provided by observations that link DNA damage to altered chromatin mobility over long distances ^31– 33^. Given that cell type–specific gene expression is regulated by productive encounters between enhancers and promoters that are often located far apart, the burst of transient DNA breaks and the dynamic formation of DNA repair factories ^25^ might also provide a protein environment that favors the affinity between enhancers and promoters to induce productive interactions.

## Non-coding eRNAs – the 3D connection

For interactions between enhancers and promoters to be manifested, increased opportunities for physical proximities will likely not be sufficient without a degree of attraction between the participating chromatin fibers. Thus, many different regions of the genome may collide with each other but will not interact in a physical and functional sense, even transiently. Conversely, other regions that display affinity to each other—by CTCF ^34^ or YY1 ^21^ pairing, for example—will have the potential to form metastable complexes. Although the inner working of this process is not well understood, there are several links to non-coding transcripts, which have been observed to be a common feature of most active enhancers ^35^. The observation that enhancers can produce such transcripts, termed eRNAs ^35^, is in retrospect not surprising. The reasons are twofold: First, the chromatin marks characterizing promoters can also be found within enhancer regions ^4^; second, the sequence complexity characterizing a functional promoter is low to occur on average every few kilobase pairs in the mammalian genome. What is more surprising is that such eRNAs appear, in some instances, to be responsible for establishing the chromatin loops between enhancers and promoters. For example, it was shown that the reduction of eRNA expression within a distally positioned colon-specific super-enhancer reduced its interaction with the *MYC* gene ^36^. However, beyond this and a few other examples, it remains to be determined whether eRNAs are more generally a consequence of the transcriptionally permissive environment at active enhancer regions rather than being directly involved in the establishment of enhancer-promoter contacts.

## The link to CTCF – a master regulator

Although CTCF has traditionally been assigned to function as a master regulator of the genome via its function as a chromatin insulator protein ^37^, it was realized early on that its functions also included transcriptional activation ^38^. For example, CTCF can interact with the large subunit of the RNA polymerase II complex, and CTCF binding sites can function as promoters of transcription ^39^. Moreover, while a wild-type CTCF binding site at the *XIST* promoter was associated with activation of *XIST* and hence X chromosome inactivation in female human cells, a naturally occurring mutation at this site led to loss of both CTCF binding and *XIST* transcription, leading to skewed patterns of X inactivation ^40^. These observations are in keeping with the more recent findings that CTCF binding sites are frequently found in enhancer regions ^41^. Two not mutually exclusive scenarios explaining such data can be envisioned: CTCF binding sites might promote transcription of eRNAs or participate in the communication between enhancers and promoters (or do both). This latter point has usually been associated with the ability of CTCF to recruit the cohesin complex to its binding sites ^14^. However, the absence of a complete overlap in the functions of these factors shows the existence of CTCF-independent recruitment of cohesin complexes to enhancers ^21, 41^. It is also known that both proximal and distal CTCF binding sites can directly interact with each other in the context of chromatin insulators to prevent variegated gene expression ^42^. Similarly, enhancers associated with CTCF binding sites have been shown to have a more stable interaction with promoters: The deletion of a key CTCF binding site led to transcriptional variation in Th2 cells, suggesting that a CTCF-mediated metastable chromatin loop underlies homogenous expression ^43^. Superimposed on these processes, CTCF binding sites can interact with each other to establish topologically associated domains (TADs) of chromatin ^34, 44, 45^ by attenuating the ability of enhancers to access genes in flanking TADs ^46^.

## How can the 3D functions of CTCF be regulated?

Although the affinity between CTCF and its binding sites can generally be controlled by DNA methylation states ^47^, the rate of turnover of such features is likely too slow to enable dynamic adjustments to changing environmental cues. A more plausible principle was provided in one early study demonstrating that transcription through a CTCF binding site was sufficient to transiently evict CTCF from chromatin ^48^. More recently, it was demonstrated that a lineage-specific enhancer could be activated by transcription through a CTCF binding site ^49^. Intriguingly, this process resulted in the relocation of the Bcl11b enhancer from the repressive compartment at the nuclear periphery to enable the subsequent targeting of this enhancer to distal promoters, essential for T-cell differentiation ^49^. It is also possible that CTCF-mediated connections between chromatin fibers can be modulated by post-translational modifications of CTCF. For example, it was shown early on that poly(ADP-ribosy)lation ^50^ and SUMOylation ^51^ of CTCF regulated its ability to confer insulator functions. The link to poly(ADP-ribosy)lated CTCF prompted the realization that CTCF binds to and activates PARP1 to influence not only the organization of chromatin networks in *cis* and in *trans* but also the oscillating transcription of circadian genes ^23^. Such information bears on the observation that PARP1 binds active promoters ^24^ to potentially set up interaction patterns with CTCF-containing enhancers. Given the role of PARP1 in DNA repair, such a scenario might also facilitate repair of any programmed DNA breaks at promoters and enhancers. It remains to be established whether other post-translational modifications, such as phosphorylation and acetylation, play a role in the CTCF/PARP1-mediated organization of the genome and to what extent this principle collaborates with other factors, such as YY1 and cohesin, to effectuate the transcriptional process.

## Is the gene looking for the enhancer or vice versa?

Given that active marks often increase the mobility of chromatin fibers ^22, 31, 32^, it is conceivable that both enhancer and promoter regions actively roam the nuclear space to increase their chances for contact. Strictly speaking, all regulatory DNA sequences including a distant enhancer (or enhancers) can be considered to be part of a gene, but for the sake of simplicity, a “gene”—typically a protein-coding transcription unit—is treated here as a separate entity. Nevertheless, the observations that such contacts are enriched within TADs ^44, 45^ show that local physical constraints increase the preferences for proximal interactions. This makes sense, as this chromatin organization restricts the employment of enhancer regions positioned either in distant TADs on the same chromosomes or in *trans*, on other chromosomes ^46, 52^. Thus, perturbations of TAD boundaries might promote expression patterns unsuitable for proper development or for maintaining cell type–specific transcription or for both ^53^. From these perspectives, the question of whether it is the enhancer or the gene that drives their interactions might seem moot. However, this deduction is at odds for at least some developmentally important genes, such as the *HOX* clusters, which are positioned in inter-TAD boundaries ^54, 55^. As enhancers in flanking TADs do not readily communicate over such borders ^54^, it is conceivable that it is the gene that searches for enhancers in either of the flanking TADs ^54, 55^. This scenario has also been borne out for *MYC*, which forms a dynamic network with enhancers in both of the flanking TADs ^56^. Importantly, in comparisons of the dynamic importance of the baits, *MYC* constitutes by far the most important and central node in a local gene-enhancer network derived from colon cancer cells ^56^. Moreover, with the ultra-sensitive Nodewalk technique to analyze very small samples, it was demonstrated that *MYC* stochastically interacted with enhancers in either of the flanking TADs in a mutually exclusive manner ^56^.

## Is there a role for enhancer chromatin hubs?

It has frequently been observed in large cell populations that enhancers extensively communicate with each other to form chromatin hubs ^52, 57– 59^. Although such structures have not yet been documented in single nuclei, they have been interpreted as evidence that enhancers collaborate in targeting gene promoters ^52, 57– 59^. This issue is particularly relevant in instances with spatially clustered, multiple enhancer elements spanning tens or hundreds of kilobase pairs to form super-enhancers, which have often evolved in the vicinity of cell fate–determining genes ^60^. Although it is certainly feasible to conclude the existence of enhancer–enhancer interactions to form a virtual network in large cell populations, the stochastic and dynamic character of chromatin fiber movements may be at odds with the idea that multiple and simultaneous enhancer networks would survive sufficiently long to target specific promoters in individual nuclei. Accordingly, single-cell analyses using DNA fluorescence *in situ* hybridization (FISH) to label regulatory regions identified to interact with each other by the 4C-seq technique are consistent with the view that chromatin networks are organized by “dates” rather than “parties” ^61^. The interpretation that regulatory elements within super-enhancers do not necessarily collaborate to active neighboring genes but instead increase the likelihood for transcriptional activation has also been borne out. Thus, with the Nodewalk technique, it has been demonstrated that *MYC* interacts with a preferential subset of regulatory elements within its oncogenic super-enhancer in stochastic manners ^56^. However, it cannot be ruled out that a metastable enhancer-promoter loop at a gene poised for transcription but not yet active is able to attract another enhancer to provide the final trigger of transcriptional activation. This possibility is finding support with the discovery of the so-called “fail-safe” or “split” enhancers that represent spatially separated enhancer units that need to be simultaneously active for the induction of highly specific transcriptional regulation ^62^. At another level, one enhancer might seed active marks on distal or proximal regions containing dormant enhancers to set up super-enhancers and indirectly promote transcription. Although they do not specifically involve enhancers, there are precedents for long-range chromatin loops affecting epigenetic states: A regulatory region binding CTCF and flanking the *H19* gene not only prevents DNA methylation of a distal regulatory region it interacts with in *cis* in somatic cells ^63^ but also is able to transfer epigenetic marks in *trans* to interacting regions during a specific developmental window of mouse spermatogonia ^61^.

## Beyond current enhancer models

**Figure 1.**
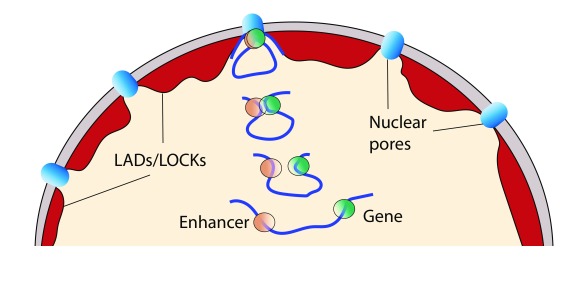
Hypothetical model of the influence of the nuclear architecture on a specific set of enhancer-promoter interactions. The dynamic juxtaposition of enhancers and genes to the nuclear periphery/nuclear pore is accompanied by increased opportunities for their interactions. The lamina-associated domains (LADs) ^82^ and large organized chromatin K9 modifications (LOCKs) ^83^ represent a robustly inactivated compartment that is perceived to increase the physical constraints on the mobility of chromatin fibers.

The vast majority of recent models attempting to describe enhancer functions in 3D present these with little or no reference to the nuclear architecture. Beyond the enigmatic task of visualizing how enhancers and genes physically communicate in 3D, we remain largely ignorant as to how and where these interactions occur in the 3D space of the nucleus. The techniques visualizing such features either do not have the resolution to detect interactions (3D DNA FISH) or cannot reliably assess the influence of the nuclear architecture on chromatin networks (“C” derived techniques) ^6^. Nonetheless, without a detailed 3D perspective, we run the risk of missing the final pieces of the puzzle with respect to what brings these regions together, the dynamics of their interactions, and how they are regulated. This is exemplified by transcription factories, which consist of several genes being simultaneously transcribed in permissive compartments located at the interface between chromosomes and the inter-chromosomal space ^64, 65^. Although such information suggests that enhancers and genes within TAD units need to exit the physical constraints provided by the core structure of chromosomes, it also raises a number of key questions pertaining to the inner workings of transcription factory formations. Are rapidly-turning-over transcription factories organized by dynamic enhancer networks that attract promoters of genes to become activated ^66^, or do they simply result from physical constraints transiently promoting co-transcription of genes that are proximal to each other in the 3D space ^67, 68^? This issue is further ****compounded by real-time analyses of chromosome movements in relation to the nuclear architecture showing that the mobility of chromatin fibers is decreased when approaching the nuclear periphery or nucleoli ^69– 71^. This feature, which is transient and hence not epigenetic in character, is likely an effect of the physical constraints provided by the microenvironment of these structural hallmarks. Thus, opportunities for interactions between the colorectal super-enhancer and the *MYC* gene might increase in a manner directly proportional to their distance from the nuclear periphery ( Figure 1). Therefore, it is likely that structural features within the nucleus can both promote or antagonize (or both) enhancer-gene interactions depending on the structural context. This interpretation is reinforced by the observations that although the nuclear periphery is frequently linked to repressive micro-domains ^72, 73^, association of chromatin to the nuclear pore complex involves both repressed and active states ^73, 74^.

## Gene gating and enhancers

More than three decades ago, it was postulated that the juxtaposition of active genes to the nuclear pore would facilitate the export of the transcript ^75^. This prophetic prediction, termed gene gating, has largely been borne out in lower eukaryotes, such as yeast, but is less well understood in mammalian cells ^76^. However, in the yeast genome, in contrast to the animal genome, long-range enhancers cannot be found ^77^. Moreover, the volume of the yeast nucleus is more than 100-fold smaller than the mammalian counterpart ^78^, implying that any mechanism to gate a mammalian gene to the nuclear pore might have to negotiate these differences. Nonetheless, it is now well established that several nucleoporins that participate in the formation of nuclear pore complexes associate with active chromatin ^79^. Indeed, it has been observed that some nucleoporins, such as NUP93 and NUP153, not only bind enhancers and promoters of developmentally important genes ^79, 80^ but also influence their expression ^79^. Although this information would suggest that nucleoporins might facilitate enhancer-nuclear pore associations, this deduction is complicated by the frequent finding that nucleoporins are often found in the nucleoplasm ^81^. This information suggests that some nucleoporins might have one function in the nucleoplasm to promote enhancer-promoter interactions and another at the nuclear membrane to participate in nuclear export/import. Conversely, it is also possible that enhancers and promoters binding nucleoporins are being recruited to the nuclear pores to enhance the export of processed transcripts. In this scenario, factors that might mediate such a function, such as ELYS ^84^, would need to collaborate with processes negotiating torsional stress induced by enhanced chromatin mobility. Therefore, it is of considerable interest that ELYS has been shown to contribute to genomic stability in mouse intestinal epithelial progenitor cells by promoting DNA repair ^85^. As enhancer- *MYC* promoter proximities increase when they approach the nuclear periphery (Scholz, Sumida, Diettrich Malled de Lima, Zhao, Barthiya, Sifakis, Göndör and Ohlsson, unpublished), we propose that the nucleoporin-enhancer link mediates the recruitment of active genes to the nuclear pore to enhance the export of a subset of nuclear transcripts ( Figure 1). The observations that the DNA damage response alters chromatin mobility and might facilitate the recruitment of transcriptional units to the nuclear pores for subsequent repair in yeast and *Drosophila* ^31– 33^ reinforce the link between enhancers, gene activation, and nuclear pores. However, such a principle remains speculative for mammalian cells for which similar data are not yet available.

## Perturbed nuclear architecture and enhancer functions

The aberrant nuclear architecture of cancer cells, such as the increased number of nucleoli, has long been observed by pathologists and has been used for diagnosis and prognosis. However, what such changes mean in functional terms is not clear—a conclusion further aggravated by intra-tumor heterogeneities to render any cause-and-effect relationship tenuous. Nonetheless, some key observations have shown that epimutations typically targeting larger chromatin domains might influence the nuclear architecture. This is exemplified by LOCKs (large organized chromatin K9 modifications) that tend to localize at the nuclear periphery ^83^ ( Figure 1). The anchoring of a chromatin region to the nuclear periphery is, with few exceptions, considered to be associated with either induced silencing or maintenance of silenced states in somatic cells ^83, 86^. The loss of such regions leads to unscheduled and variable gene expression and has been linked with poor prognosis of patients with cancer ^83^. Therefore, it has been proposed that tumor-specific chromatin folding might undermine differentiation in part by perturbing the dynamics and specificity of enhancer-promoter communication in tumor progenitor cells ^87^. The nucleolus is gaining traction for similar reasons: Apart from representing a key bottle neck for cancer cell proliferation—by controlling the production of ribosomes and hence the potential for protein production—the nucleolus has the peculiar feature that active rRNA genes are inside the nucleolus while inactive rRNA genes loop out to seed perinucleolar heterochromatin ^88^. Interestingly, this compartment is converted from a transcriptionally repressive to a transcriptionally permissive environment in cancer cells of patients of poor prognosis ^89^. Therefore, we speculate that perturbations of heterochromatin around the nucleolus are propagated throughout the cancer cell nucleus to create havoc on regulatory enhancer networks that might require structural features of the normal nuclear architecture for their maintenance.

## Some unresolved questions

During the last decade, a vast amount of information has uncovered that diverse enhancer functions involve multiple features of the epigenome and its folding in 3D. It has been and remains a formidable task to chisel out how these and many other features collaborate within the single nucleus to provide the high-precision transcriptional regulation that is required during normal development. Key unresolved questions that are still only scratching the surface address how enhancers and genes find each other in the nuclear space and how the stability of these interactions is regulated. Moreover, it is still unknown whether the stability of enhancer-promoter interactions is a factor in deciding the efficiency of RNA polymerase loading on to the promoter and, if so, whether the enhancer needs to stably interact with promoters not yet active but poised for transcription. Would the maintained presence of an enhancer-gene loop facilitate re-initiation of transcription? In one instance involving rRNA genes, for example, it was demonstrated that the enhancer/promoter and terminator regions make contact to form a “ribomotor” to enhance levels of rRNA transcription ^90^. Similarly, it remains to be established whether the affinity between enhancers and promoters is metastable in itself to require an active process of disruption by post-translational modifications, for example. Or will the dynamic mobility of other chromatin regions within TADs compromise such stabilities by generating long-range torsional stress or by DNA repair-induced local stiffening of the chromatin fiber or both ^91^? Can an enhancer-promoter loop generate secondary but transient marks to establish a higher affinity between the RNA polymerase complex and target promoters? Finally, the existence of transcription factories is generally acknowledged though often ignored because of a lack of detailed knowledge regarding their composition. Do these represent side products resulting from physical constraints, or do they facilitate coordination of expression?

## Concluding remarks and outlook

Owing to advances in high-throughput sequencing, dogmas are now being formed in the chromatin biology field at a neck-breaking speed. Although the emergence of high-throughput data has revolutionized our understanding of the enhancer-mediated transcriptional process, it should be pointed out that current chromatin technologies display severe limitations in either resolution or sensitivity as well as in their abilities to correctly quantify key features of chromatin biology. It is now possible to readily study movements of individual loci in real time using, for example, the SNP-CLING ^92^ and CARGO dCas9 ^93^ imaging techniques, and aside from clever combinations of ChIP-seq and Hi-C approaches ^94^ to generate a detailed mapping of radial chromatin features, such as chromatin marks and compaction at a high resolution in 3D in single cells using the chromatin *in situ* proximity (ChrISP) technique ^95, 96^. However, these techniques will likely not be sufficient to understand the mechanism of enhancer action. This two-tier progress of the chromatin field is further complicated by the realization that biological processes impinging on chromatin features are very dynamic ^97^ and are governed by stochastic principles ^9^. Thus, there is likely a need to characterize a large number of single cells for chromatin structures to generate statistically relevant perceptions of their varying 3D conformations. The potential influence of computer simulations that can be generated from such data ^98^ should not be underestimated. When a role of the nuclear architecture in promoting or antagonizing enhancer-gene interactions is considered, there is the additional requirement to combine analysis of 3D epigenomic features within single cells and score for such features in relation to nuclear structure and gene activity. To resolve the huge challenges lying ahead of this field, there is likely a need to develop a new generation of chromatin techniques.

## References

[1] Schaffner W: Enhancers, enhancers - from their discovery to today's universe of transcription enhancers. *Biol Chem.* 2015;396(4):311–27. 10.1515/hsz-2014-0303 25719310

[2] Coppola CJ C Ramaker R Mendenhall EM: Identification and function of enhancers in the human genome. *Hum Mol Genet.* 2016;25(R2):R190–R197. 10.1093/hmg/ddw216 27402881

[3] Pennacchio LA Bickmore W Dean A: Enhancers: five essential questions. *Nat Rev Genet.* 2013;14(4):288–95. 10.1038/nrg3458 23503198 PMC4445073

[4] Calo E Wysocka J: Modification of enhancer chromatin: what, how, and why? *Mol Cell.* 2013;49(5):825–37. 10.1016/j.molcel.2013.01.038 23473601 PMC3857148

[5] Zaret KS Carroll JS: Pioneer transcription factors: establishing competence for gene expression. *Genes Dev.* 2011;25(21):2227–41. 10.1101/gad.176826.111 22056668 PMC3219227

[6] Göndör A Ohlsson R: Chromosome crosstalk in three dimensions. *Nature.* 2009;461(7261):212–7. 10.1038/nature08453 19741702

[7] Shlyueva D Stampfel G Stark A: Transcriptional enhancers: from properties to genome-wide predictions. *Nat Rev Genet.* 2014;15(4):272–86. 10.1038/nrg3682 24614317

[8] Meng H Bartholomew B: Emerging Roles of Transcriptional Enhancers In Chromatin Looping And Promoter-Proximal Pausing Of RNA Polymerase II. *J Biol Chem.* 2017; pii: jbc.R117.813485. 10.1074/jbc.R117.813485 29187597 PMC6130961

[9] Fiering S Whitelaw E Martin DI: To be or not to be active: the stochastic nature of enhancer action. *Bioessays.* 2000;22(4):381–7. 10.1002/(SICI)1521-1878(200004)22:4<381::AID-BIES8>3.0.CO;2-E 10723035

[10] Atlasi Y Stunnenberg HG: The interplay of epigenetic marks during stem cell differentiation and development. *Nat Rev Genet.* 2017;18(11):643–58. 10.1038/nrg.2017.57 28804139

[11] Müeller-Storm HP Sogo JM Schaffner W: An enhancer stimulates transcription in *trans* when attached to the promoter via a protein bridge. *Cell.* 1989;58(4):767–77. 10.1016/0092-8674(89)90110-4 2548735

[12] Yan J Chen SA Local A: Histone H3 lysine 4 monomethylation modulates long-range chromatin interactions at enhancers. *Cell Res.* 2018;28(2):204–220. 10.1038/cr.2018.1 29313530 PMC5799818

[13] Allen BL Taatjes DJ: The Mediator complex: a central integrator of transcription. *Nat Rev Mol Cell Biol.* 2015;16(3):155–66. 10.1038/nrm3951 25693131 PMC4963239

[14] Baranello L Kouzine F Levens D: CTCF and cohesin cooperate to organize the 3D structure of the mammalian genome. *Proc Natl Acad Sci U S A.* 2014;111(3):889–90. 10.1073/pnas.1321957111 24398527 PMC3903212

[15] Kagey MH Newman JJ Bilodeau S: Mediator and cohesin connect gene expression and chromatin architecture. *Nature.* 2010;467(7314):430–5. 10.1038/nature09380 20720539 PMC2953795

[16] Ohlsson R: Gene expression: The coherent Mediator. *Nature.* 2010;467(7314):406–7. 10.1038/467406a 20864988

[17] Guenther MG Levine SS Boyer LA: A chromatin landmark and transcription initiation at most promoters in human cells. *Cell.* 2007;130(1):77–88. 10.1016/j.cell.2007.05.042 17632057 PMC3200295

[18] Apostolou E Thanos D: Virus Infection Induces NF-kappaB-dependent interchromosomal associations mediating monoallelic *IFN-beta* gene expression. *Cell.* 2008;134(1):85–96. 10.1016/j.cell.2008.05.052 18614013

[19] Engreitz JM Haines JE Perez EM: Local regulation of gene expression by lncRNA promoters, transcription and splicing. *Nature.* 2016;539(7629):452–5. 10.1038/nature20149 27783602 PMC6853796

[20] Rusk N: Towards a dynamic 3D genome. *Nat Meth.* 2018;15:31 10.1038/nmeth.4544

[21] Weintraub AS Li CH Zamudio AV: YY1 Is a Structural Regulator of Enhancer-Promoter Loops. *Cell.* 2017;171(7):1573–1588.e28. 10.1016/j.cell.2017.11.008 29224777 PMC5785279

[22] Tumbar T Sudlow G Belmont AS: Large-scale chromatin unfolding and remodeling induced by VP16 acidic activation domain. *J Cell Biol.* 1999;145(7):1341–54. 10.1083/jcb.145.7.1341 10385516 PMC2133171

[23] Zhao H Sifakis EG Sumida N: PARP1- and CTCF-Mediated Interactions between Active and Repressed Chromatin at the Lamina Promote Oscillating Transcription. *Mol Cell.* 2015;59(6):984–97. 10.1016/j.molcel.2015.07.019 26321255

[24] Krishnakumar R Gamble MJ Frizzell KM: Reciprocal binding of PARP-1 and histone H1 at promoters specifies transcriptional outcomes. *Science.* 2008;319(5864):819–21. 10.1126/science.1149250 18258916

[25] Puc J Aggarwal AK Rosenfeld MG: Physiological functions of programmed DNA breaks in signal-induced transcription. *Nat Rev Mol Cell Biol.* 2017;18(8):471–6. 10.1038/nrm.2017.43 28537575 PMC5854152

[26] Ju BG Lunyak VV Perissi V: A topoisomerase IIbeta-mediated dsDNA break required for regulated transcription. *Science.* 2006;312(5781):1798–802. 10.1126/science.1127196 16794079

[27] Baranello L Wojtowicz D Cui K: RNA Polymerase II Regulates Topoisomerase 1 Activity to Favor Efficient Transcription. *Cell.* 2016;165(2):357–71. 10.1016/j.cell.2016.02.036 27058666 PMC4826470

[28] Perillo B Ombra MN Bertoni A: DNA oxidation as triggered by H3K9me2 demethylation drives estrogen-induced gene expression. *Science.* 2008;319(5860):202–6. 10.1126/science.1147674 18187655

[29] Zuchegna C Aceto F Bertoni A: Mechanism of retinoic acid-induced transcription: histone code, DNA oxidation and formation of chromatin loops. *Nucleic Acids Res.* 2014;42(17):11040–55. 10.1093/nar/gku823 25217584 PMC4176188

[30] Puc J Kozbial P Li W: Ligand-dependent enhancer activation regulated by topoisomerase-I activity. *Cell.* 2015;160(3):367–80. 10.1016/j.cell.2014.12.023 25619691 PMC4422651

[31] Seeber A Gasser SM: Chromatin organization and dynamics in double-strand break repair. *Curr Opin Genet Dev.* 2017;43:9–16. 10.1016/j.gde.2016.10.005 27810555

[32] Caridi PC Delabaere L Zapotoczny G: And yet, it moves: nuclear and chromatin dynamics of a heterochromatic double-strand break. *Philos Trans R Soc Lond B Biol Sci.* 2017;372(1731): pii: 20160291. 10.1098/rstb.2016.0291 28847828 PMC5577469

[33] Agarwal P Miller K: Chromatin dynamics and DNA repair.In: Göndör A, editor. *Chromatin regulation and dynamics* Elsevier;2017;275–302. 10.1016/B978-0-12-803395-1.00011-3

[34] Rao SS Huntley MH Durand NC: A 3D map of the human genome at kilobase resolution reveals principles of chromatin looping. *Cell.* 2014;159(7):1665–80. 10.1016/j.cell.2014.11.021 25497547 PMC5635824

[35] Liu F: Enhancer-derived RNA: A Primer. *Genomics Proteomics Bioinformatics.* 2017;15(3):196–200. 10.1016/j.gpb.2016.12.006 28533025 PMC5487531

[36] Xiang JF Yin QF Chen T: Human colorectal cancer-specific *CCAT1*-L lncRNA regulates long-range chromatin interactions at the *MYC* locus. *Cell Res.* 2014;24(5):513–31. 10.1038/cr.2014.35 24662484 PMC4011346

[37] Phillips JE Corces VG: CTCF: master weaver of the genome. *Cell.* 2009;137(7):1194–211. 10.1016/j.cell.2009.06.001 19563753 PMC3040116

[38] Ohlsson R Lobanenkov V Klenova E: Does CTCF mediate between nuclear organization and gene expression? *Bioessays.* 2010;32(1):37–50. 10.1002/bies.200900118 20020479 PMC6375297

[39] Chernukhin I Shamsuddin S Kang SY: CTCF interacts with and recruits the largest subunit of RNA polymerase II to CTCF target sites genome-wide. *Mol Cell Biol.* 2007;27(5):1631–48. 10.1128/MCB.01993-06 17210645 PMC1820452

[40] Pugacheva EM Tiwari VK Abdullaev Z: Familial cases of point mutations in the XIST promoter reveal a correlation between CTCF binding and pre-emptive choices of X chromosome inactivation. *Hum Mol Genet.* 2005;14(7):953–65. 10.1093/hmg/ddi089 15731119

[41] Zuin J Dixon JR van der Reijden MI: Cohesin and CTCF differentially affect chromatin architecture and gene expression in human cells. *Proc Natl Acad Sci U S A.* 2014;111(3):996–1001. 10.1073/pnas.1317788111 24335803 PMC3903193

[42] Guibert S Zhao Z Sjölinder M: CTCF-binding sites within the *H19* ICR differentially regulate local chromatin structures and cis-acting functions. *Epigenetics.* 2012;7(4):361–9. 10.4161/epi.19487 22415163

[43] Ren G Jin W Cui K: CTCF-Mediated Enhancer-Promoter Interaction Is a Critical Regulator of Cell-to-Cell Variation of Gene Expression. *Mol Cell.* 2017;67(6):1049–1058.e6. 10.1016/j.molcel.2017.08.026 28938092 PMC5828172

[44] Nora EP Goloborodko A Valton AL: Targeted Degradation of CTCF Decouples Local Insulation of Chromosome Domains from Genomic Compartmentalization. *Cell.* 2017;169(5):930–944.e22. 10.1016/j.cell.2017.05.004 28525758 PMC5538188

[45] Dixon JR Selvaraj S Yue F: Topological domains in mammalian genomes identified by analysis of chromatin interactions. *Nature.* 2012;485(7398):376–80. 10.1038/nature11082 22495300 PMC3356448

[46] Smith EM Lajoie BR Jain G: Invariant TAD Boundaries Constrain Cell-Type-Specific Looping Interactions between Promoters and Distal Elements around the *CFTR* Locus. *Am J Hum Genet.* 2016;98(1):185–201. 10.1016/j.ajhg.2015.12.002 26748519 PMC4716690

[47] Mukhopadhyay R Yu W Whitehead J: The binding sites for the chromatin insulator protein CTCF map to DNA methylation-free domains genome-wide. *Genome Res.* 2004;14(8):1594–602. 10.1101/gr.2408304 15256511 PMC509268

[48] Lefevre P Witham J Lacroix CE: The LPS-induced transcriptional upregulation of the chicken lysozyme locus involves CTCF eviction and noncoding RNA transcription. *Mol Cell.* 2008;32(1):129–39. 10.1016/j.molcel.2008.07.023 18851839 PMC2581490

[49] Isoda T Moore AJ He Z: Non-coding Transcription Instructs Chromatin Folding and Compartmentalization to Dictate Enhancer-Promoter Communication and T Cell Fate. *Cell.* 2017;171(1):103–119.e18. 10.1016/j.cell.2017.09.001 28938112 PMC5621651

[50] Yu W Ginjala V Pant V: Poly(ADP-ribosyl)ation regulates CTCF-dependent chromatin insulation. *Nat Genet.* 2004;36(10):1105–10. 10.1038/ng1426 15361875

[51] MacPherson MJ Beatty LG Zhou W: The CTCF insulator protein is posttranslationally modified by SUMO. *Mol Cell Biol.* 2009;29(3):714–25. 10.1128/MCB.00825-08 19029252 PMC2630690

[52] Ing-Simmons E Seitan VC Faure AJ: Spatial enhancer clustering and regulation of enhancer-proximal genes by cohesin. *Genome Res.* 2015;25(4):504–13. 10.1101/gr.184986.114 25677180 PMC4381522

[53] Dowen JM Fan ZP Hnisz D: Control of cell identity genes occurs in insulated neighborhoods in mammalian chromosomes. *Cell.* 2014;159(2):374–87. 10.1016/j.cell.2014.09.030 25303531 PMC4197132

[54] Rodríguez-Carballo E Lopez-Delisle L Zhan Y: The *HoxD* cluster is a dynamic and resilient TAD boundary controlling the segregation of antagonistic regulatory landscapes. *Genes Dev.* 2017;31(22):2264–81. 10.1101/gad.307769.117 29273679 PMC5769770

[55] Berlivet S Paquette D Dumouchel A: Clustering of tissue-specific sub-TADs accompanies the regulation of *HoxA* genes in developing limbs. *PLoS Genet.* 2013;9(12):e1004018. 10.1371/journal.pgen.1004018 24385922 PMC3873244

[56] Sumida N Sifakis E Scholz BA: The ultra-sensitive Nodewalk technique identifies stochastic from virtual, population-based enhancer hubs regulating MYC in 3D: Implications for the fitness of cancer cells.Posted on-line Mar. 27, 2018 at *BioRxiv.* 2018; Posted on-line Mar. 27, 2018. 10.1101/286583

[57] Patrinos GP de Krom M de Boer E: Multiple interactions between regulatory regions are required to stabilize an active chromatin hub. *Genes Dev.* 2004;18(12):1495–509. 10.1101/gad.289704 15198986 PMC423198

[58] Gavrilov AA Razin SV: Spatial configuration of the chicken alpha-globin gene domain: immature and active chromatin hubs. *Nucleic Acids Res.* 2008;36(14):4629–40. 10.1093/nar/gkn429 18621783 PMC2504291

[59] Markenscoff-Papadimitriou E Allen WE Colquitt BM: Enhancer interaction networks as a means for singular olfactory receptor expression. *Cell.* 2014;159(3):543–57. 10.1016/j.cell.2014.09.033 25417106 PMC4243057

[60] Whyte WA Orlando DA Hnisz D: Master transcription factors and mediator establish super-enhancers at key cell identity genes. *Cell.* 2013;153(2):307–19. 10.1016/j.cell.2013.03.035 23582322 PMC3653129

[61] Sandhu KS Shi C Sjölinder M: Nonallelic transvection of multiple imprinted loci is organized by the *H19* imprinting control region during germline development. *Genes Dev.* 2009;23(22):2598–603. 10.1101/gad.552109 19933149 PMC2779760

[62] Smith E Shilatifard A: Enhancer biology and enhanceropathies. *Nat Struct Mol Biol.* 2014;21(3):210–9. 10.1038/nsmb.2784 24599251

[63] Kurukuti S Tiwari VK Tavoosidana G: CTCF binding at the *H19* imprinting control region mediates maternally inherited higher-order chromatin conformation to restrict enhancer access to *Igf2*. *Proc Natl Acad Sci U S A.* 2006;103(28):10684–9. 10.1073/pnas.0600326103 16815976 PMC1484419

[64] Cremer T Cremer M: Chromosome territories. *Cold Spring Harb Perspect Biol.* 2010;2(3):a003889. 10.1101/cshperspect.a003889 20300217 PMC2829961

[65] Smeets D Markaki Y Schmid VJ: Three-dimensional super-resolution microscopy of the inactive X chromosome territory reveals a collapse of its active nuclear compartment harboring distinct Xist RNA foci. *Epigenetics Chromatin.* 2014;7:8. 10.1186/1756-8935-7-8 25057298 PMC4108088

[66] Cai S Lee CC Kohwi-Shigematsu T: SATB1 packages densely looped, transcriptionally active chromatin for coordinated expression of cytokine genes. *Nat Genet.* 2006;38(11):1278–88. 10.1038/ng1913 17057718

[67] Hilbert L Sato Y Kimura H: Transcription establishes microenvironments that organize euchromatin. *bioRxiv.* 2017 10.1101/234112

[68] Cisse II Izeddin I Causse SZ: Real-time dynamics of RNA polymerase II clustering in live human cells. *Science.* 2013;341(6146):664–7. 10.1126/science.1239053 23828889

[69] Chubb JR Boyle S Perry P: Chromatin motion is constrained by association with nuclear compartments in human cells. *Curr Biol.* 2002;12(6):439–45. 10.1016/S0960-9822(02)00695-4 11909528

[70] Marshall WF: Gene expression and nuclear architecture during development and differentiation. *Mech Dev.* 2003;120(11):1217–30. 10.1016/j.mod.2003.05.001 14623434

[71] Masusawa N Urata Y Yagi K: Constrained, Random, and Independent Motion of Texas-Red-labeled Chromatin in Living Interphase PtK2 Cells. *Acta Histochem Cytochem.* 2000;33(6):419–27. 10.1267/ahc.33.419

[72] Shimi T Pfleghaar K Kojima S: The A- and B-type nuclear lamin networks: microdomains involved in chromatin organization and transcription. *Genes Dev.* 2008;22(24):3409–21. 10.1101/gad.1735208 19141474 PMC2607069

[73] Cremer T Cremer M Hübner B: The 4D nucleome: Evidence for a dynamic nuclear landscape based on co-aligned active and inactive nuclear compartments. *FEBS Lett.* 2015;589(20 Pt A):2931–43. 10.1016/j.febslet.2015.05.037 26028501

[74] Pascual-Garcia P Capelson M: Nuclear pores as versatile platforms for gene regulation. *Curr Opin Genet Dev.* 2014;25:110–7. 10.1016/j.gde.2013.12.009 24632227

[75] Blobel G: Gene gating: a hypothesis. *Proc Natl Acad Sci U S A.* 1985;82(24):8527–9. 10.1073/pnas.82.24.8527 3866238 PMC390949

[76] Burns LT Wente SR: From hypothesis to mechanism: uncovering nuclear pore complex links to gene expression. *Mol Cell Biol.* 2014;34(12):2114–20. 10.1128/MCB.01730-13 24615017 PMC4054283

[77] Barberis A Pearlberg J Simkovich N: Contact with a component of the polymerase II holoenzyme suffices for gene activation. *Cell.* 1995;81(3):359–68. 10.1016/0092-8674(95)90389-5 7736588

[78] Dion V Gasser SM: Chromatin movement in the maintenance of genome stability. *Cell.* 2013;152(6):1355–64. 10.1016/j.cell.2013.02.010 23498942

[79] Capelson M Liang Y Schulte R: Chromatin-bound nuclear pore components regulate gene expression in higher eukaryotes. *Cell.* 2010;140(3):372–83. 10.1016/j.cell.2009.12.054 20144761 PMC2821818

[80] Ibarra A Benner C Tyagi S: Nucleoporin-mediated regulation of cell identity genes. *Genes Dev.* 2016;30(20):2253–8. 10.1101/gad.287417.116 27807035 PMC5110992

[81] Maeshima K Iino H Hihara S: Nuclear pore formation but not nuclear growth is governed by cyclin-dependent kinases (Cdks) during interphase. *Nat Struct Mol Biol.* 2010;17(9):1065–71. 10.1038/nsmb.1878 20711190

[82] Bickmore WA van Steensel B: Genome architecture: domain organization of interphase chromosomes. *Cell.* 2013;152(6):1270–84. 10.1016/j.cell.2013.02.001 23498936

[83] Timp W Feinberg AP: Cancer as a dysregulated epigenome allowing cellular growth advantage at the expense of the host. *Nat Rev Cancer.* 2013;13(7):497–510. 10.1038/nrc3486 23760024 PMC4636434

[84] Gillespie PJ Khoudoli GA Stewart G: ELYS/MEL-28 chromatin association coordinates nuclear pore complex assembly and replication licensing. *Curr Biol.* 2007;17(19):1657–62. 10.1016/j.cub.2007.08.041 17825564 PMC2267255

[85] Gao N Davuluri G Gong W: The nuclear pore complex protein Elys is required for genome stability in mouse intestinal epithelial progenitor cells. *Gastroenterology.* 2011;140(5):1547–55.e10. 10.1053/j.gastro.2011.01.048 21315719 PMC3282118

[86] Robson MI de Las Heras JI Czapiewski R: Constrained release of lamina-associated enhancers and genes from the nuclear envelope during T-cell activation facilitates their association in chromosome compartments. *Genome Res.* 2017;27(7):1126–38. 10.1101/gr.212308.116 28424353 PMC5495065

[87] Feinberg AP Koldobskiy MA Göndör A: Epigenetic modulators, modifiers and mediators in cancer aetiology and progression. *Nat Rev Genet.* 2016;17(5):284–99. 10.1038/nrg.2016.13 26972587 PMC4888057

[88] Guetg C Santoro R: Formation of nuclear heterochromatin: the nucleolar point of view. *Epigenetics.* 2012;7(8):811–4. 10.4161/epi.21072 22735386 PMC3427276

[89] Pollock C Huang S: The perinucleolar compartment. *Cold Spring Harb Perspect Biol.* 2010;2(2):a000679. 10.1101/cshperspect.a000679 20182614 PMC2828281

[90] Németh A Guibert S Tiwari VK: Epigenetic regulation of TTF-I-mediated promoter-terminator interactions of rRNA genes. *EMBO J.* 2008;27(8):1255–65. 10.1038/emboj.2008.57 18354495 PMC2367401

[91] Herbert S Brion A Arbona JM: Chromatin stiffening underlies enhanced locus mobility after DNA damage in budding yeast. *EMBO J.* 2017;36(17):2595–608. 10.15252/embj.201695842 28694242 PMC5579376

[92] Maass PG Barutcu AR Shechner DM: Spatiotemporal allele organization by allele-specific CRISPR live-cell imaging (SNP-CLING). *Nat Struct Mol Biol.* 2018;25(2):176–84. 10.1038/s41594-017-0015-3 29343869 PMC5805655

[93] Gu B Swigut T Spencley A: Transcription-coupled changes in nuclear mobility of mammalian cis-regulatory elements. *Science.* 2018;359(6379):1050–5. 10.1126/science.aao3136 29371426 PMC6590518

[94] Paulsen J Sekelja M Oldenburg AR: Chrom3D: three-dimensional genome modeling from Hi-C and nuclear lamin-genome contacts. *Genome Biol.* 2017;18(1):21. 10.1186/s13059-016-1146-2 28137286 PMC5278575

[95] Chen X Shi C Yammine S: Chromatin *in situ* proximity (ChrISP): single-cell analysis of chromatin proximities at a high resolution. *BioTechniques.* 2014;56(3):117–8, 120–4. 10.2144/000114145 24641475

[96] Chen X Yammine S Shi C: The visualization of large organized chromatin domains enriched in the H3K9me2 mark within a single chromosome in a single cell. *Epigenetics.* 2014;9(11):1439–45. 10.4161/15592294.2014.971633 25482057 PMC4623470

[97] Voss TC Hager GL: Dynamic regulation of transcriptional states by chromatin and transcription factors. *Nat Rev Genet.* 2014;15(2):69–81. 10.1038/nrg3623 24342920 PMC6322398

[98] Bianco S Chiariello AM Annunziatella C: Predicting chromatin architecture from models of polymer physics. *Chromosome Res.* 2017;25(1):25–34. 10.1007/s10577-016-9545-5 28070687

